# Editorial for the Special Issue on 2D Nanomaterials Processing and Integration in Miniaturized Devices

**DOI:** 10.3390/mi12030254

**Published:** 2021-03-02

**Authors:** Candido Fabrizio Pirri, Matteo Cocuzza

**Affiliations:** 1 Department of Applied Science and Technology (DISAT), Politecnico di Torino, C.so Duca degli Abruzzi 24, 10129 Turin, Italy; 2 Center for Sustainable Future Technologies, Istituto Italiano di Tecnologia, Via Livorno 60, 10144 Turin, Italy; 3 Institute of Materials for Electronics and Magnetism, IMEM-CNR, Parco Area delle Scienze 37/A, 43124 Parma, Italy

Initially considered little more than a scientific curiosity, the family of 2D nanomaterials has become increasingly popular over the last decade. Starting from the undisputed progenitor, i.e., graphene, which to date has reached a technological maturity and a critical mass of knowledge to allow envisaging multiple applications in real manufacturing, numerous other materials have progressively been added to this family: transition metal dichalcogenides, boron nitride, metal oxide nanosheets, MXenes, layered double hydroxides, etc. Similarly, the range of applications under study and development has expanded, integrating and taking advantage of the unique properties of 2D nanostructured materials: sensors, optical applications, photovoltaics, touch screens, catalysis, filtration and exploitation as fillers to modulate the mechanical, electrical and chemical properties of the host matrices. Such a plethora of material/application combinations, in addition to the obvious technical requirements related to the synthesis of materials and the optimization of purity, composition, morphology and yield, also presents significant technological challenges for the corresponding processing, patterning and above all integration into systems and devices of higher dimensionality for the real exploitation of their unique properties. The present Special Issue is then focused on such last topics, collecting eight research papers and one review article dealing with MoS_2_ [1], graphene [2,3,4] and other 2D nanomaterials integrated onto several kinds of materials and structures (nanogap [1], porous silicon [5], silicon carbide [6]) and for different applications (photoluminescence [1], ink-jet printing [7], optics and plasmonics [2,8], lubrication [3], innovative patterning [4], biomedicine [9]).

In particular, Yang et al. [1] proposed an innovative solution to bypass the limitation of the atomic thickness of monolayer MoS_2_ hindering its optical absorption and emission in view of optoelectronic applications. By integrating monolayer MoS_2_ onto nanometer wide gold nanogap arrays, it was possible to exploit the associated plasmon resonance, thus enhancing photoluminescence by a factor 20x, and thus paving the way for successive applications in photodetectors, sensors and emitters. Zhang J. et al. [2] studied another hybrid metamaterial consisting in metal–graphene. Such a coupling gives rise to a sharper Fano resonance than the pure graphene and it is, moreover, adjustable as a function of the number of graphene layers. Plasmonic sensing applications with extremely high sensitivity in the mid-infrared range are envisioned. Zhang L. et al. [3] showcase an interesting and relatively unconventional application for graphene, i.e., lubrication for structural ceramics. In particular, Si_3_N_4_/ Si_3_N_4_ sliding pair tribological properties (lubrication and cooling) have been investigated through the addition of different weight contents of graphene to a base lubricating oil. Relevant results have been obtained and an explanation about the lubricating improvement mechanism was provided. In the last paper involving graphene, Verna et al. [4] showed an original and straightforward process, based on the lift-off of the catalyst seed layer, to pattern few-layer graphene. The direct chemical vapor deposition of graphene on the patterned seed layer guaranteed high quality of the resulting 2D material and a 10 µm patterning resolution was demonstrated.

Volovlikova et al. [5] analyzed the effect of illumination intensity and p-dopant concentration on the dissolution properties of silicon for its photo-assisted etching with no external bias or metals to produce porous silicon. A thorough characterization was performed, providing valuable data for the control of porous silicon thickness and porosity. Ruffino et al. [6] provided a valuable basic study on the growth and coalescence characteristics of a nanoscale-thick bimetallic film of Au/Pd on a silicon carbide substrate. The kinetic of the growth process was studied from the initial 3D clustering to the final continuous rough thin film formation. Xiao et al. [7] performed a full comparison, with special attention to electrical resistivity and adhesion, between ink-jet-printed silver thin films based on nanoparticle ink and metal–organic decomposition ink cured by two different approaches, that is to say, UV exposure and heat-assisted approaches. Cao et al. [8] fabricated (through a combination of lithography and nanoimprint technology) and characterized a flexible 3D display film element consisting of two integrated structures of a microimage array and microlens array.

Finally, in their review article, Coluccio et al. [9] revised and critically described nanoscale transport phenomena and biomedical applications of different emerging electrochemical devices whose working principle relies on the interaction between ions and conductive polymers.

Last, but not least, we would like to thank all the contributing authors and all the involved reviewers for their precious contribution to the assembly and quality of this Special Issue.
